# Human Pluripotent Stem Cell-Derived Cardiac Cells: Application in Disease Modeling, Cell Therapy, and Drug Discovery

**DOI:** 10.3389/fcell.2021.655161

**Published:** 2021-04-01

**Authors:** Juan Huang, Qi Feng, Li Wang, Bingying Zhou

**Affiliations:** ^1^Shenzhen Key Laboratory of Cardiovascular Disease, Fuwai Hospital, Chinese Academy of Medical Sciences, Shenzhen, China; ^2^State Key Laboratory of Cardiovascular Disease, Fuwai Hospital, National Center for Cardiovascular Diseases, Chinese Academy of Medical Sciences and Peking Union Medical College, Beijing, China

**Keywords:** pluripotent stem cell, cell therapy, drug discovery, cardiac cell, disease modeling

## Abstract

Cardiac diseases are the leading cause of deaths worldwide; however, to date, there has been limited progress in the development of therapeutic options for these conditions. Animal models have been the most extensively studied methods to recapitulate a wide variety of cardiac diseases, but these models exhibit species-specific differences in physiology, metabolism and genetics, which lead to inaccurate and unpredictable drug safety and efficacy results, resulting in drug attrition. The development of human pluripotent stem cell (hPSC) technology in theory guarantees an unlimited source of human cardiac cells. These hPSC-derived cells are not only well suited for traditional two-dimensional (2-D) monoculture, but also applicable to more complex systems, such as three-dimensional (3-D) organoids, tissue engineering and heart on-a-chip. In this review, we discuss the application of hPSCs in heart disease modeling, cell therapy, and next-generation drug discovery. While the hPSC-related technologies still require optimization, their advances hold promise for revolutionizing cell-based therapies and drug discovery.

## Introduction

Although cardiac diseases, such as myocardial infarction and heart failure, have been the leading cause of deaths worldwide, few drugs are being approved yearly compared with many other disease area, resulting in a huge gap between clinical need and drug development (Fordyce et al., 2015). The regenerative capacity of the heart is quickly lost during mammalian postnatal development. Pathological insults, such as ischemia, almost invariably lead to irreversible cardiac cell loss, which poses the greatest challenge to the treatment of cardiac diseases. In the past decades, rapid progress in human pluripotent stem cell (hPSC) technology enabled the generation of major cardiac cell types, thus unlocking new possibilities to treat patients with the most debilitating forms of heart disease using cell-based therapies (Mummery et al., 2012; Protze et al., 2019; Williams and Wu, 2019). To date, several studies have shown that transplantation of hPSC-derived cardiomyocytes holds great promise for attenuating cardiac dysfunction and reducing consequent fibrotic scarring (Gao et al., 2018; Liu et al., 2018).

Animal models are arguably the most widely used method to model disease onset and progression, providing valuable mechanistic insights in an *in vivo* setting. However, the extrapolation of translatable data from animals data to guide the treatment of human heart disease has been difficult due to considerable species differences (Matsa et al., 2014). hPSCs can potentially bridge this transitional gap by providing an unlimited source of human cardiac cells for biomedical research and drug discovery (Matsa et al., 2014). Protocols that enable the generation of cardiomyocyte subtypes such as ventricular, atrial, and sinoatrial pacemaker cells, as well as non-myocyte cell types including endothelial cells and fibroblasts have been developed (Mummery et al., 2012; Weng et al., 2014; Lee et al., 2017; Protze et al., 2017; Williams and Wu, 2019; Zhang et al., 2019). These highly enriched populations of specific cardiomyocyte subtypes facilitate disease modeling and drug testing by recognizing cellular, molecular and functional heterogeneity within the heart. Further, considering the spatial complexity of the heart, hPSC-based 3-D multicellular systems, including human heart organoids (Mills et al., 2017; Giacomelli et al., 2020), engineered heart tissues (EHTs) (Mannhardt et al., 2016), and heart-on-chip models (Zhang et al., 2016) have been shown to more accurately predict human cardiac biology and pathophysiology (Sharma et al., 2020). Incorporation of electrophysiology, epigenomics, transcriptomics, proteomics, metabolomics and imaging techniques will equip these hPSC-based platforms with additional tools to accomplish patient-specific disease modeling and personalized drug response screening.

**FIGURE 1:**
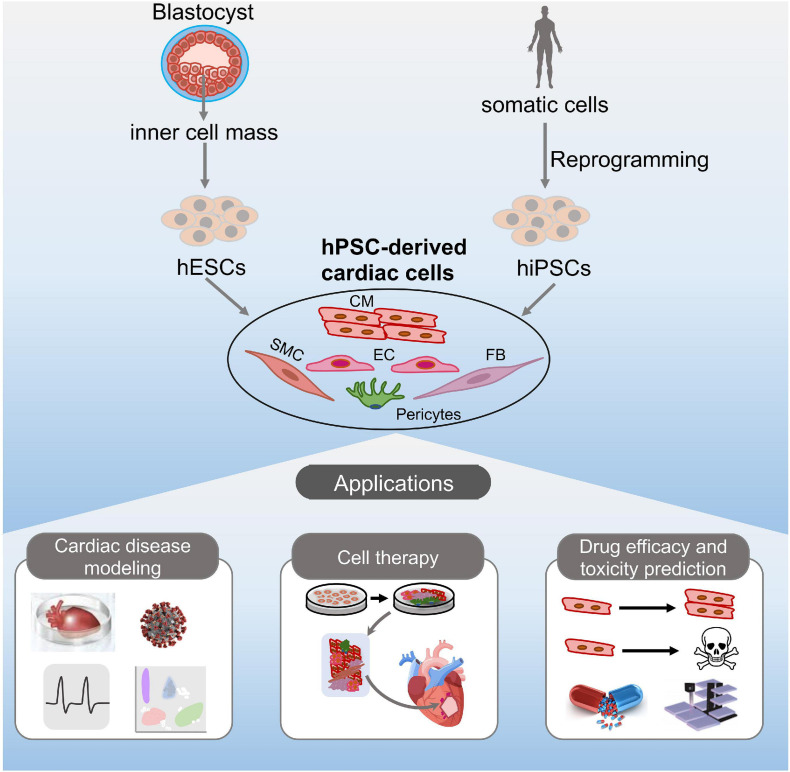
Potential applications of hPSC-derived cardiac cells. hESCs or hiPSCs can differentiate into various cardiac cell types *in vitro*, including cardiomyocytes, endothelial cells, fibroblasts, smooth muscle cells, and pericytes. The resulting cells can be potentially used for disease modeling, cell therapy, and drug discovery.

In this review, we summarize the current progress of the application of hPSCs in cardiac disease and cell therapies (Figure 1). We also highlight the application of available hPSC platforms for the pharmacologic evaluation and cardiac safety assessment of drugs (Figure 1).

## Cardiac Disease Modeling Using hPSCs

Modeling heart disease is central to the understanding of the pathological processes. Conventional models of cardiovascular disease rely mostly on animals. However, the inherent differences between species render elucidation of human heart pathology unexpectedly difficult (Savoji et al., 2019). Therapeutics with promising results in animals often failed to show any improvement in clinical trials. Therefore, human-derived disease models bear the unique advantage to more faithfully represent human disease, thus potentially providing a more refined comprehension of disease mechanisms, paving the way to new therapeutic options (Zhao et al., 2020).

One of the major causes of cardiac disorders is genetic mutations (Dell’Era et al., 2015). The advent of iPSC technology provides a powerful means to model genetic cardiac diseases by using somatic cells directly from patients. In the past decade, various genetic cardiac disorders have been successfully modeled using iPSCs. For example, Timothy syndrome is caused by a missense mutation in L-type calcium channel Ca_*v*_1.2 that leads to excess Ca^2+^ influx, prolonged action potential and irregular contraction. Yazawa et al. (2011) reported the generation of iPSC-CMs derived from patients with Timothy syndrome, and these cells demonstrated irregular electrical activity and abnormal Ca^2+^ signaling similar to the cardiac phenotype found in the patients. iPSCs have also been used to model LEOPARD syndromes with *PTPN11* gene mutation (Carvajal-Vergara et al., 2010), long QT syndromes with a mutation in *KCNQ1* (Friedrichs et al., 2013), arrhythmogenic right ventricular dysplasia with *PKP2* mutations (Kim et al., 2013), and dilated cardiomyopathy with *TTN* mutations (Hinson et al., 2015).

One potential drawback of such application is that differences in the genetic background among cell lines may conceal the true phenotype induced by a single mutation. To address this challenge, the introduction of genome-editing techniques, such as clustered regularly interspaced short palindromic repeats- (CRISPR-) associated protein 9 (Cas9) system, help to generate isogenic iPSC lines, and allows researchers to study the precise effect of a mutation on the onset or progression of cardiac diseases while avoiding confounding genetic factors (Seeger et al., 2017; Lam and Wu, 2018). By correcting mutations or insertions, CRISPR/Cas9 further facilitates the elucidation of the causal role of the mutation. For example, CRISPR/Cas9 was successfully used to identify SCN5A as a causative mutation of arrhythmogenic right ventricular cardiomyopathy (ARVC), and corrected cells showed normal channel activity (Te Riele et al., 2017).

**FIGURE 2:**
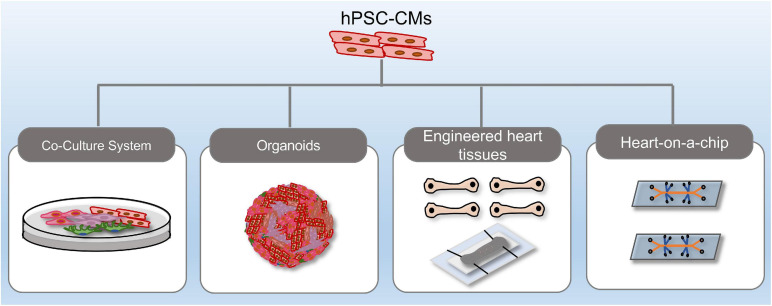
Recent methodological advances in hPSC-derived complex platforms. hPSC-derived platforms have grown in complexity from simple, two-dimensional cultures into multi-lineage co-cultures, heart organoids, engineered heart tissues, and heart-on-a-chip systems.

The heart is composed of multiple cell types, including cardiomyocytes and non-cardiomyocytes, and the intricate crosstalk and multifaceted regulation among cells are central to heart homeostasis and disease. In this perspective, co-culture of hiPSC-derivatives, 3-D organoids, EHTs and microfluidic organ-chips exhibit distinct advantages over mono-lineage cultures (Figure 2). Using relatively immature iPSC-CMs to represent adult-onset disorders remains challenging. Recent studies demonstrated that co-culture of fibroblasts, epicardial cells, or endothelial cells with hiPSC-CMs enhanced the maturity of hiPSC-CMs, which improved modeling of cardiac diseases (Bargehr et al., 2019; Giacomelli et al., 2020). Still, there are some limitations regarding regular 2-D culture, such as lacking a 3D extracellular matrix and a defined auxotonic load. 3-D cardiac constructs including organoids and EHTs overcome these limitations and improve representation of the in *vivo* cardiovascular environment. Cardiac organoids are generated from hPSCs that self-assemble and -organize into complex native-like organ structures (Richards et al., 2020). Voges et al. (2017) reported successful generation of an AMI model in human ESC-derived cardiac organoids. Cryoinjury induced local tissue damage, while the adjacent cells remained viable. Functional evaluation showed that tissue regeneration accompanied by functional recovery was seen 14-days post-injury (Voges et al., 2017). Another hiPSC-derived cardiac organoids that cooperate an oxygen-diffusion gradient and that are stimulated with neurotransmitter noradrenaline was shown to recapitulate the infarcted, border and remote zones of myocardial infarction in the human heart, with concurrent modeling of the hallmarks of AMI, including metabolic shifts, fibrosis and aberrant calcium handling (Richards et al., 2020).

Engineered heart tissues can be generated by mixing hPSC-derived cardiomyocytes with extracurricular matrix components such as fibrinogen, collagen or Matrigel (Breckwoldt et al., 2017). The generation of EHTs also requires a casting mold that determines the 3-D shape of the heart tissue, and a support structure that provides mechanical restraint of the developing heart tissue (Eder et al., 2016). EHTs are well suited to evaluate the effects of mechanical stimulation, and therefore can mimic afterload enhancement-induced disease conditions such as aortic valve stenosis, chronic hypertension, and cardiac hypertrophy (Hirt et al., 2012; Tzatzalos et al., 2016). Since EHTs are little heart muscles, they allow measurements of all essential parameters of heart muscle function, including, but not restricted to, contractile force, conduction velocity, beating rate, rhythm, diastolic tension, passive tension, and intracellular Ca^2+^ transients (Hirt et al., 2014). Besides, EHTs are also compatible with histological analyses, protein detection, and sequencing techniques, such as single-cell RNA-sequencing.

The development of 3D stamping and bioprinting techniques provides scaffolds to generate heart-on-a-chip. EHTs and heart-on-a-chip methods allowed modeling of specific cardiac diseases, including Barth-syndrome-associated cardiomyopathy (Wang et al., 2014), Duchenne muscular dystrophy (Long et al., 2018) and primary hypertension-induced left ventricular hypertrophy (Zhao et al., 2019). Zhao et al. (2019) took advantage of organ-on-a-chip engineering and organoid self-assembly to generate mature ventricular tissue, and to perform electrical conditioning for up to 8 months, allowing modeling of chronic, polygenic conditions. With the generation of a wide variety of human organ-on-a-chip models, great efforts have been made to integrate multisensory systems. Zhang et al. (2017) reported two multiorgan models, liver- and-heart-on-a-chip and heart-liver-cancer-on-a-chip, for automated and continual *in situ* monitoring of organoid behaviors.

Recently, hPSCs have also been proven quite useful in the study of COVID-19-related heart disorders. To date, this pandemic has led to more than 121 million infections and 2.6 million deaths worldwide. Apart from respiratory complications, COVID-19 is also known to induce cardiac complications, including myocardial injury, arrhythmias, acute coronary syndrome, which are major indicators of poor prognosis (Nishiga et al., 2020; Zheng et al., 2020). Since one of the major receptors for COVID-19 is not recognized by the virus in mice, hPSCs were rapidly recognized as an invaluable tool to address this problem, and has already yielded important insight into virus-host interactions, immune responses and the cytokine storm (Yang et al., 2020; Yiangou et al., 2020). Combined with various biochemical, cellular, molecular and genetic studies, these newly developed hPSCs-cardiac models will likely contribute much more to uncovering key mechanisms and therapeutic strategies of COVID-19-induced cardiac damage (Yiangou et al., 2020).

## hPSC-Based Cell Therapy

Aside from mechanistic studies of cardiac disease, replenishing damaged myocardium with healthy cells (i.e., cell therapy) was another hotly pursued area of application. Transplantation of hESC-derived cardiomyocytes (hESC-CMs) was found to improve cardiac function in postinfarct rats and pigs (Kehat et al., 2004; Caspi et al., 2007; Laflamme et al., 2007). More importantly, Chong et al. (2014) showed that exogenously transplanted hESC-CMs remuscularized the infarcted area in a non-human primate model. However, hESCs may not be the best option for clinical treatment due to ethical concerns and immune rejection. Ethical concerns arise from their origins, since these cells are isolated from the inner cell mass of the human embryo, leading to the destruction of the latter. Immune rejection occurs due to allogenic transplantation of cells. By contrast, iPSC technology, in which pluripotent stem cells are directly reprogrammed from the same patient’s somatic cells, effectively circumvents the ethical issues associated with the use of ESCs. Additionally, iPSCs are considered autologous, and are believed not to require immunosuppression. Gene editing in hiPSC further helps to reverse disease phenotype by correction of the pathogenic mutation or variant, raising the possibility of personalized therapies for autologous stem cell transplantation.

Injecting hPSC-derived cardiac cells into the myocardium to replace dead cells appears to be a very straightforward concept. However, in practical terms, controlling the exact cell number, limited efficiency in cell delivery to target sites, and batch differences, all pose great challenges to the application of hPSC-based cell therapy (Matsuura et al., 2013; Guo et al., 2020). With the development of tissue engineering, stem cell-derived cell sheets gradually showed their superiority over direct injection in heart tissue repair (Matsuura et al., 2013). Kawamura et al. (2012) showed that highly pure (almost 90%) hiPSC-CMs sheets were able to attenuate left ventricular remodeling, increase neovascularization as well as inhibit fibrosis 8 weeks after cell transplantation into 12-week porcine models of myocardial infarction. The same group demonstrated that the combined use of cell sheets and the omental flap technique was beneficial even in treating severe heart failure (Kawamura et al., 2017). Most recently, it was reported that the transplantation of clinically relevant dimensions (4 cm × 2 cm × 1.25 mm) of human cardiac muscle patches significantly improved left ventricular function and reduced infarct size in infarcted swine (Gao et al., 2018).

Mechanistically, transplantation of hiPSCs-derived cardiac cells may provide a favorable microenvironment for pre-existing cells in the infarcted zone to proliferate, thus preventing serious post-MI events (Ye et al., 2014). The mechanisms by which injected cells exert these effects are still not fully clear. It has been shown that only a limited number of transplanted cells can differentiate and retain in the host myocardium after delivery, suggesting that the beneficial effects of cell therapy are mediated by the activation of paracrine pathways which leads to endogenous regeneration (Tachibana et al., 2017). The autocrine and paracrine factors facilitate angiogenesis, promote vascularization, attenuate fibrosis and relieve inflammation. Still, the clinical translation of hPSC-based cell therapy is facing a major problem, i.e., the immaturity of hPSC-derived cells, which can cause life-threatening arrhythmia, and teratoma formation (Cambria et al., 2017). Even vascularized cell sheets co-cultured with different types of cells exhibit an immature phenotype. As a source of *de novo* cardiomyocytes, hPSC-CMs have so far yielded only a short-term improvement in cardiac function ranging from weeks to months. Therefore, more advanced strategies to induce maturation, vascularization, and to improve durable cardiac function recovery are worth investigation.

## hPSCs-Based Drug Discovery

Human pluripotent stem cell technology is also widely used in cardiovascular drug discovery, providing pharmacologic and toxicologic predictions. Yazawa et al. (2011) found that roscovitine, a compound that increases the voltage-dependent inactivation of CaV1.2, rescued the cardiomyocyte phenotypes in Timothy syndrome. Using hPSC-derived cardiomyocytes and organoids, compounds that promoted human heart muscle cell proliferation, but minimally affected heart rhythm and contractility, were identified (Mills et al., 2019). Although the identified compounds displayed poor pharmacokinetic properties, and were only maintained at pro-proliferative doses *in vivo* for a short period, they led to the elucidation of mevalonate pathway important for cardiomyocyte proliferation. Most recently, Theodoris et al. (2020) reported that a network-based screen in iPSC-derived cells revealed therapeutic candidates for a common form of heart disease involving the aortic valves, suggesting that the combined use of network-based screening, iPSC technique, and machine learning may represent an effective approach for drug discovery. Together, these inspiring hPSC-based drug studies offer meaningful pipelines to identify drug candidates that may lead to new therapeutic options for heart disease.

Unexpected cardiotoxicity is a major cause of drug attrition and drug withdrawal from the market (Laverty et al., 2011). Traditional methods of preclinical cardiac safety evaluation mainly rely on animal models, which tend to be expensive, low-throughput, and exhibit species differences in cardiac physiology (Mercola et al., 2013). Alternative methods to identify cardiotoxic drugs involve the heterologous expression of cardiac ion channels in non-cardiac cells. However, these non-cardiac cells lack CM-specific structural components such as sarcomeres, and inhibition of specific ionic currents alone cannot accurately measure the effects of drug candidates, which could miss potential arrhythmogenic effects or generate false-positive readings. Other cells, such as healthy and primary human CMs, may be used for preclinical evaluation of drug cardiotoxicity. However, sample access and cellular abundance may be prohibitive for its use in an industrial setting. Regulatory agencies around the globe, including US FDA, European EMA, Health Canada, and Japan NIHS, have recognized the lack of standardized assessment of cardiac safety, and thus encouraged the development of the Comprehensive *In Vitro* Proarrhythmia Assay (CiPA), which aims to explore the utility of hPSC-derived cardiomyocyte assays in evaluating cardiac safety and arrhythmogenesis (Sager et al., 2014). Data from recent studies support the utility of hPSC-derived cardiomyocytes for predicting drug-induced arrhythmia (Sager et al., 2014; Gintant et al., 2016; Blinova et al., 2018). Besides electrophysiology, hiPSC-CMs are also used to measure drug-induced alterations in cellular contractility and viability, thus broadening the scope of cardiac safety evaluation. Sharma et al. (2018) developed a detailed methodology to generate hiPSC-CMs and subsequently use these cells to evaluate drug-induced cardiotoxicity by using contractility and cytotoxicity assays. Using this platform, they performed cardiotoxicity screening of tyrosine kinase inhibitors, with results correlating with clinical phenotypes (Sharma et al., 2017). They also demonstrated that the observed toxicities could be ameliorated with cardioprotective insulin/IGF signaling.

Although hPSC-CMs raised hopes that this human test bed could broaden drug discovery approaches and improve preclinical drug testing, they are not identical with mature adult CMs. Important distinctions in ion channel function, gene expression, structural organization and functional responses to drugs limit their application for drug testing. Compared to standard 2D culture formats, engineered 3D heart tissues improve CM maturity, and exhibit a more physiological 3D muscle environment, longitudinal alignment, and easy access of measurements of force, which is one of the most important parameters of heart function (Eder et al., 2016). However, one major drawback of EHTs is their inability to scale up for the high-throughput screening of multiple samples in parallel. To some extent, EHTs in 24-well format or cardiac tissue miniaturization might provide possible solutions for drug testing purposes (Thavandiran et al., 2013; Eder et al., 2016).

Zhao et al. (2019) developed a scalable cardiac tissue cultivation platform that reconstructs a specific anatomic portion of the heart to facilitate more directed and accurate measurements of drug responses. “Human-heart-in-a-jar” is also a related technology, which involves embedding ventricular-like hiPSC-CMs in hydrogel to create an electromechanically coupled cardiac organoids chamber that is capable of pumping fluid. This organoid chamber is amenable to clinically relevant functional measurements, such as pressure-volume loop analysis, cardiac output, and ejection fraction (Li et al., 2018). As the field of iPSC technology continues to grow, to increase the efficiency of drug discovery and to reduce the cost, an established set of standards for the comparison of the quality of iPSC-CMs, quantification of cardiac contraction and electrophysiology, and validation of data and reproducibility, is desperately needed.

## Challenges and Future Outlook

Despite major advancements in the application of PSC technology in cardiac diseases, several challenges remain, and require further in-depth research. As the structure, signaling, metabolism, and function in immature CMs are distinct from their adult counterparts, iPSC-CMs derived disease models might not be able to accurately reflect the true disease phenotype, making it difficult to confidently assess the efficacy or toxicity of drug candidates. Recently, various studies have tried to enhance the maturity of hiPSC-CMs via different means, including using T3 hormone, metabolic maturation, 3-D construction, mechanic stress, electrical stimulation, long-term culturing, and co-culture with other cell types such as cardiac fibroblasts and endothelial cells (Ahmed et al., 2020). However, it is worthwhile to point out that these “matured” cells still differ from adult human cardiomyocytes in many aspects, and therefore require careful data interpretation. Other limitations, such as scalability and clinical compatibility, also need to be addressed before one can faithfully extrapolate findings from such models. Developing optimal methods to efficiently generate large-scale mature hPSC-CMs is therefore of high importance and priority.

Furthermore, limited blood perfusion of cells within complex structures, especially for cell patches, is another issue yet to be overcome. Otherwise, oxygen, nutrient and drug delivery, which all depend on the microvasculature, would be compromised. Simple co-culture of cardiomyocytes with endothelial cells only generates premature micro-vasculatures that are not sufficient to support perfusion, leading to the small size of cardiac organoids (i.e., 100 μm). To introduce microvascularization into cell patches, Schaefer et al. (2018) engineered a bilayer patch composed of a layer of human iPSC-CMs and a layer of human blood outgrowth endothelial cells. Implantation of this bilayer patch into infarcted rat hearts resulted in better CM survival compared to CM-only patch control. Importantly, after 4 weeks *in vivo*, the engrafted microvessels sprouted into the accompanying CM layer, and even became inosculated with the host vasculature. Qian et al. (2019) co-cultured human mesenchymal stem cells and endothelial cells in decellularized human dermal fibroblast sheets. However, without actual implantation *in vivo*, one can hardly infer the reparative effects of such patches purely based on biochemical data. Clearly, engineering heart-like tissues with intact microvasculature is still in its infancy. Once solved, *in vitro* cardiac models would be able to accommodate even more diversified functional assessments, offering greater versatility and yielding higher predictability.

## Conclusion

In this review, we outlined the current applications of hPSC-CMs in disease modeling, cell therapy and drug discovery. While hPSC technology has gained momentum in cardiac repair over the years, efforts to enhance the maturity of derived cells and to increase the complexity of tissue structure are still underway. Integrating genetic-, computer-, and bioengineering-based approaches will further empower this technique with greater precision, breadth and depth. It can be foreseen that through these interdisciplinary endeavors, hPSC technology will redefine *in vitro* cardiac modeling, promote personalized treatment, and increase the efficiency but reduce the cost of drug discovery.

## Author Contributions

All authors listed have made a substantial, direct and intellectual contribution to the work, and approved it for publication.

## Conflict of Interest

The authors declare that the research was conducted in the absence of any commercial or financial relationships that could be construed as a potential conflict of interest.
